# Spinal cord transverse section hinders Wallerian degeneration and neoangiogenesis after peripheral nerve transection: Involvement of Schwann cells' miR-134–5p

**DOI:** 10.1016/j.ibneur.2026.06.012

**Published:** 2026-06-20

**Authors:** Lingli Jiang, Fang Zhang, Fang Qi, Guangchao Xu, Shene Xiao, Ubaldo Armato, Anna Chiarini, Ilaria Dal Prà, Daniele De Santis, Chengliang Deng, Zairong Wei

**Affiliations:** a Department of Burns and Plastic Surgery, Affiliated Hospital of Zunyi Medical University, Zunyi, Guizhou, China; b Maxillofacial Surgery & Odontostomatology Section, Department of Surgery, Dentistry, Paediatrics & Gynaecology, University of Verona Medical School, Verona 37134, Italy

**Keywords:** Spinal cord injury, Peripheral nerve regeneration, Wallerian degeneration, Schwann cell, MiRNAs, MiR-134–5p

## Abstract

Patients with spinal cord injury (SCI) frequently develop chronic wounds in paralyzed limbs, underscoring the importance of neural regulation and peripheral nerve integrity for tissue repair; however, it remains unclear how concomitant SCI alters the distal responses to peripheral nerve injury (PNI) and the associated microRNA (miRNA) profile. Sprague–Dawley rats underwent sciatic nerve transection alone (PNI group) or complete spinal cord transection at L1–L2 combined with sciatic transection (SCI+PNI group), and distal sciatic nerve stumps were harvested at 0, 3, and 7 days for gross observation, H&E and toluidine blue staining, myelin basic protein (MBP) immunofluorescence, transmission electron microscopy, and CD31 immunohistochemistry to assess Wallerian degeneration and angiogenesis; miRNA expression profiles were analyzed by small RNA sequencing and validated by RT-qPCR, and the effects of miR-134–5p on rat Schwann cell were evaluated using mimics or inhibitors in Transwell migration, scratch-wound, and CCK-8 proliferation assays. In the PNI group, distal stumps exhibited marked edema, disruption of fiber architecture, rapid myelin breakdown, and a robust increase in CD31⁺ vascular rings, whereas compared with PNI, the SCI+PNI group showed milder edema and structural disruption, higher numbers of intact myelin rings, and increased MBP signal at days 3 and 7 together with a markedly smaller CD31⁺ area, indicating delayed Wallerian degeneration and reduced angiogenesis. Small RNA sequencing identified multiple differentially expressed miRNAs, with miR-134–5p and miR-142–5p significantly downregulated in the SCI+PNI group and this pattern confirmed by RT-qPCR; functionally, miR-134–5p overexpression enhanced Schwann cell migration and proliferation, while inhibition produced the opposite effects. These findings indicate that SCI attenuates distal nerve responses to PNI by delaying myelin clearance, impairing angiogenesis, and downregulating repair-promoting miRNAs such as miR-134–5p, suggesting that targeting miRNA-mediated Schwann cell regulation may provide new strategies to improve peripheral nerve and wound repair below the level of SCI.

## Highlights

• SCI delays Wallerian degeneration in distal peripheral nerve stumps.
• Angiogenesis is significantly impaired in distal peripheral nerves after SCI.
• miR-134–5p is downregulated in distal peripheral nerves following SCI combined PNI.
• miR-134–5p promotes Schwann cell migration and proliferation in vitro.
• Neural regulation is essential for the peripheral nerve microenvironment.

## 1 Introduction

Spinal cord injury (SCI) is a devastating neurological disorder that often causes permanent motor and sensory deficits below the level of the lesion. Patients with SCI frequently suffer from chronic wounds in paralyzed limbs, highlighting the importance of neural regulation in tissue repair (Martinez-Torija et al., 2025, Xu et al., 2025). Notably, accumulating evidence indicates that peripheral nerve integrity is essential in this process, as sensory and autonomic fibers release neuropeptides and neurotransmitters that modulate inflammation, angiogenesis, and cellular behavior during wound healing (Bi et al., 2025, Kiya and Kubo, 2019, Yang et al., 2024). These observations raise the question of whether SCI-induced loss of central neural input may also compromise the intrinsic regenerative capacity of peripheral nerves themselves, thereby contributing to poor tissue repair below the level of injury.

Compared with the central nervous system, peripheral nerves possess a remarkable intrinsic capacity for regeneration. Following peripheral nerve injury (PNI), distal axonal stumps undergo anterograde Wallerian degeneration, a tightly coordinated program that involves myelin breakdown, macrophage recruitment, Schwann cell de-differentiation and proliferation, and remodeling of the endoneurial microvasculature (Chen et al., 2019, Wei et al., 2024, Wu et al., 2025). These events collectively create a permissive microenvironment that supports axonal regrowth and remyelination from the proximal nerve end. Repair-phenotype Schwann cell are central to this process: they clear myelin debris, form Büngner bands that guide regenerating axons, and secrete a wide range of trophic and angiogenic factors (Wei et al., 2024, Wu et al., 2025). The timing and quality of Wallerian degeneration strongly influence regeneration success, and dysregulated inflammatory or vascular responses can hinder functional recovery. However, most mechanistic studies of Wallerian degeneration and Schwann cell biology have been performed in neurologically intact animals. How a concomitant central lesion such as SCI reshapes the distal peripheral nerve response to injury remains largely unexplored.

Recent work has shown that SCI brings about widespread systemic and peripheral alterations beyond the spinal cord itself, including chronic immune dysfunction, autonomic imbalance, and microcirculatory disturbances (Martinez-Torija et al., 2025, Wang et al., 2023). The autonomic nervous system provides innervation to peripheral nerves and their associated vasculature (Krishnan et al., 2024), and its disruption after SCI has been shown to impair vasomotor control, reduce blood flow to peripheral tissues, and alter immune cell trafficking (Yuan et al., 2019). Furthermore, Schwann cell—the principal glial cells of the peripheral nervous system—are known to respond to neural signals, and their repair functions, including de-differentiation, proliferation, and myelin clearance, are influenced by the local neural and vascular microenvironment. These changes can modify macrophage recruitment and activation, Schwann cell phenotypic switching, and endothelial-cell behavior following a PNI, thereby altering the microenvironment in which Wallerian degeneration and regeneration occur. Nevertheless, there is limited in vivo evidence directly comparing how distal peripheral nerves respond to injury in the presence or absence of a SCI. A better understanding of these interactions could help explain why tissues in paralyzed limbs exhibit poor regenerative capacity and might reveal novel molecular targets to enhance repair.

MicroRNAs (miRNAs) have emerged as crucial post-transcriptional regulators of gene expression in the peripheral nervous system. Numerous miRNAs are dynamically regulated after PNI and have been shown to influence Schwann cell myelinophagy, proliferation, migration, and myelination, as well as neuroinflammation and angiogenesis (Borger et al., 2022, Li et al., 2023, Yi et al., 2016). For example, miR−1, miR−195–5p, and others modulate Schwann cell behavior by targeting key growth-factor and transcription-factor pathways, thereby affecting axonal regeneration (Li et al., 2023, Yi et al., 2016). However, the miRNA landscape after SCI in peripheral nerve distal stumps is largely unexplored. Among candidate miRNAs, miR−134–5p and miR−142–5p have been implicated in various neurological settings, including neuropathic pain, ischemic injury, and blood–brain barrier dysfunction, where they regulate neuroinflammation, neuronal survival, and endothelial integrity (Ji et al., 2019, Wang et al., 2017, Xu et al., 2024). Their potential roles in peripheral nerve regeneration and Schwann cell biology, particularly in the context of SCI, are not yet defined.

In this study, we established a rat model in which sciatic nerve transection was performed either alone (PNI group) or combined with complete spinal cord transection at L1–L2 (SCI+PNI group). Distal sciatic nerve stumps were examined at 0, 3 and 7 days by gross observation, histology, myelin basic protein (MBP) immunofluorescence, transmission electron microscopy and CD31 immunohistochemistry to evaluate Wallerian degeneration and angiogenesis. Concurrently, small-RNA sequencing with RT-qPCR validation characterized miRNA expression profiles. Next, we assessed the effects of miR−134–5p on rat Schwann cell using gain- and loss-of-function approaches combined with migration and proliferation assays. Our results show that SCI blunts the acute response of the distal sciatic nerve stump after PNI, delays myelin clearance, and impairs angiogenesis. These changes are accompanied by the downregulation of miR−134–5p, which promotes Schwann cell migration and proliferation in vitro. Thus, our findings suggest that SCI+PNI creates a less permissive microenvironment for peripheral nerve regeneration, thereby contributing to poor tissue repair below the level of the injury.

## 2 Materials and methods

### 2.1 Animals and ethics statement

This study was conducted in strict accordance with the recommendations in the Guide for the Care and Use of Laboratory Animals of the National Institutes of Health. The protocol was approved by the Institutional Animal Care and Use Committee (IACUC) of Zunyi Medical University (Approval No. KLLY (A)−2019–009). All experimental procedures complied with the ARRIVE guidelines 2.0 (Animal Research: Reporting of In Vivo Experiments), the U.K. Animals (Scientific Procedures) Act 1986, and the EU Directive 2010/63/EU for animal experiments.

A total of 54 male Sprague-Dawley rats aged 6–8 weeks (weighing 220–250 g) were purchased from Tengxin Biological Company (Chongqing, China). Male rats were selected to eliminate potential variability caused by the estrous cycle on nerve regeneration. Animals were adaptively housed for 1 week in a specific pathogen-free environment under standard conditions (12-hour light/dark cycle; 20–24 °C; 45%–65% humidity) with ad libitum access to water and standard rodent chow. All efforts were made to minimize the number of animals used and their suffering, strictly adhering to the "3 R" principles (Replacement, Reduction, and Refinement).

### 2.2 Randomisation and blinding

Animals were randomly assigned to groups using a random-number table by an investigator not involved in outcome assessment. Outcome assessment and data analysis were performed blinded to group allocation. Specifically, investigators quantifying histology, immunostaining, TEM and performing RNA-seq bioinformatic analyses, RT-qPCR data processing and statistics were blinded using coded sample IDs. The surgeon could not be blinded due to the nature of the procedures. Group codes were revealed only after completion of the final analyses.

### 2.3 Grouping and model establishment

A total of 54 SD rats were allocated to two experimental groups: PNI and SCI+PNI. Animals were euthanized and distal sciatic nerve stumps were collected at 0, 3 and 7 days post-surgery. The experimental unit was a single animal. The planned sample size was n = 9 animals per group per time point (total n = 54), which was determined based on previous studies. All surgical procedures were performed under aseptic conditions. Anesthesia was induced with isoflurane (4%) and maintained at 1.5% in 100% oxygen delivered at 0.8 L/min using a small-animal anesthesia system. Adequate anesthetic depth was confirmed by loss of the pedal withdrawal reflex and stable respiratory rate. Body temperature was maintained at ∼37℃ using a warming pad throughout the procedure, and ophthalmic ointment was applied to prevent corneal drying. To minimize perioperative pain, animals received analgesia with buprenorphine, 0.05 mg/kg, administered subcutaneously pre-operatively and every 12 h for 72 h.

**Fig. 1:**
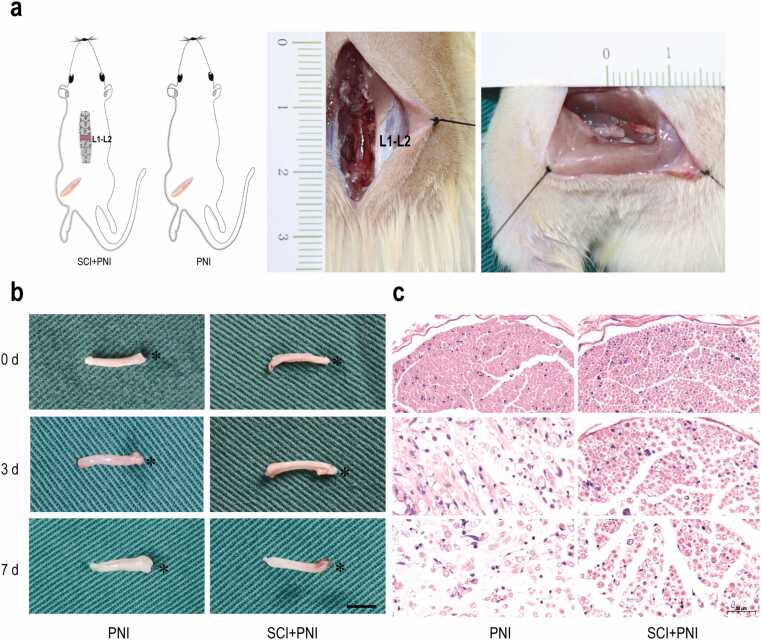
Experimental design and blunted distal sciatic stump response after PNI combined with SCI (a) Schematic of experimental groups and surgical procedures in Sprague–Dawley rats. The SCI+PNI group underwent complete transection of the spinal cord between L1 and L2 combined with sciatic nerve transection. The PNI group received sciatic nerve transection alone. After spinal cord transection, a gelatin sponge was placed between the cord ends. After sciatic nerve transection, proximal and distal stumps were fixed with 10–0 nylon sutures to maintain a ∼2 mm gap. (b) Gross appearance of the distal sciatic nerve stump (marked by “*”) at day 0 and at 3 and 7 days after surgery. At day 0, distal stumps appeared normal in both groups. At day 3, the PNI group showed edema and softening, whereas the SCI+PNI group showed no visible edema. At day 7, pronounced edema was present in the PNI group, while the SCI+PNI group exhibited only softening without overt edema. n = 3 per group per time point, scale bar = 5 mm. (c) H&E staining of the distal sciatic nerve stump at different time points. At day 0, nerve fibers in both groups were regularly and compactly arranged. At day 3, fibers in the PNI group were disorganized with evident edema, whereas fibers in the SCI+PNI group remained orderly but less densely packed. At day 7, normal fiber architecture was lost in the PNI group, whereas the SCI+PNI group still retained the basic nerve fiber structure, although fibers were loosely arranged. n = 3 per group per time point, scale bar = 50 μm.

In the PNI group, animals underwent unilateral sciatic nerve transection only, whereas in the SCI+PNI group, animals received spinal cord transection at the L1–L2 level combined with sciatic nerve transection (Fig. 1a). Briefly, anesthetized rats were placed in the prone position, and a midline skin incision was made over the most prominent part of the spinal column (L1–L2). Although the sciatic nerve originates from spinal segments L4–L6, L1–L2 transection was selected because it lies rostral to these segments, thereby ensuring complete interruption of descending supraspinal pathways to the lower limbs. This level induces high paraplegia while preserving respiratory function, and has been widely used to study peripheral consequences of spinal cord injury. The skin and underlying tissues were dissected until the spinal cord was exposed, then completely transected. The stumps were trimmed to create a ∼5 mm gap, and a gelatin sponge was inserted to control bleeding and keep separate the two ends of the cord. For sciatic nerve transection, a skin incision parallel to the pelvic notch was made in the lower limb. The sciatic nerve was exposed by blunt dissection, and the mid-portion was cut to create a 2 mm gap between the proximal and distal stumps, which were then fixed in place.

Following surgery, animals were placed on a warming pad until fully recovered from anesthesia and returned to clean cages with softened food and accessible water. Animals were monitored at least twice daily for the first 72 h and thereafter daily for general condition, body weight, wound integrity, signs of pain/distress (e.g., piloerection, reduced activity, vocalization), and self-mutilation/autotomy. For SCI animals, assisted bladder expression was performed 3 times daily, and cage bedding was changed frequently to maintain hygiene. Humane endpoints were predefined as > 20% body weight loss, severe wound infection/dehiscence, persistent self-mutilation, inability to access food/water, or moribund state, and animals meeting these criteria were euthanized immediately.

At 0, 3, and 7 days after surgery, animals were euthanized by isoflurane overdose (≥5% in oxygen until respiratory arrest) followed by cervical dislocation as a secondary method to ensure death. Death was confirmed by absence of respiration and heartbeat and lack of reflexes. Approximately 1 cm of distal sciatic nerve stump was then harvested for gross examination and subsequent analyses.

### 2.4 Hematoxylin–eosin (H&E) staining

Harvested nerve tissues were fixed in 10% formaldehyde solution to preserve histomorphology and antigenicity. The tissues were embedded in paraffin and cut into 5 μm-thick longitudinal sections along the nerve axis. Sections were deparaffinized in xylene and rehydrated through graded ethanol solutions (100%, 95%, 90%, 80%). Next, they were stained with H&E for 5 min and rinsed with running tap water to stop the reaction. Finally, sections were dehydrated, dried and mounted, and structural changes of the nerve tissue were observed under a light microscope.

### 2.5 Toluidine blue staining

Section deparaffinization and rehydration were performed as described for H&E staining. Sections were stained with toluidine blue solution for 5 min, briefly differentiated in 1% glacial acetic acid, and rinsed with tap water to stop staining. Next, they were dried and cleared in xylene for 5 min. Finally, sections were mounted with neutral gum and observed under a light microscope. For quantitative analysis, an intact myelin sheath was defined as a toluidine blue-stained structure exhibiting a clear, continuous, closed ring-like profile without evidence of vacuolation, fragmentation, or lamellar separation. The number of intact myelin sheaths was counted in 3–5 randomly selected high-power fields per section.

### 2.6 Immunofluorescence and immunohistochemical staining

Section pretreatment was the same as for H&E staining. Sections were incubated with 3% hydrogen peroxide for 15 min to inactivate endogenous peroxidase. After washing, antigen retrieval was performed by heating in citrate buffer (pH 6.0, 0.01 mol/L). To reduce non-specific binding, sections were blocked with normal goat serum for 30 min at 37 ℃. Primary antibodies against MBP (1:200, Servicebio, China) or CD31 (1:400, Abcam, United Kingdom) were applied and sections were incubated overnight at 4 ℃ in a humidified chamber. After 12 h, sections were washed and incubated with the appropriate secondary antibody (1:2000, Abcam, United Kingdom) for 60 min at 37 ℃ in the dark. Nuclei were counterstained with DAPI for 5 min. After rinsing with phosphate buffer, sections were mounted with an anti-fade medium and examined under a fluorescence microscope. MBP immunofluorescence intensity was quantified using Fiji ImageJ software. In 3–5 randomly selected high-power fields per section, the integrated density of MBP^+^ signal was measured under identical acquisition parameters, and the mean intensity per field was calculated. The CD31^+^ area was quantified using Fiji ImageJ software. The total immune positive area was expressed in μm² and the number of CD31^+^ vascular rings, defined as endothelial‑lined structures with a clearly identifiable lumen, was manually counted in each field to assess microvessel formation.

### 2.7 Transmission electron microscopy

Fresh sciatic nerve tissues taken at 0, 3 and 7 days after modeling from rats belonging to both groups were cut into blocks of approximately 1 mm × 1 mm × 1 mm. Samples were first fixed in EM fixative (Servicebio, China) and then post-fixed in 1% osmic acid (Servicebio, China). After fixation, tissues were dehydrated through a graded ethanol series (50%, 70%, 80%, 90%, 95%, 100% I, 100% II) followed by 100% acetone (acetone I and II), 15 min at each step. They were then infiltrated with a mixture of acetone and 812 embedding resin (SPI, USA) for 2–4 h and embedded in pure 812 resin. Ultrathin sections (60–80 nm) were cut using an ultramicrotome (Leica, China) and stained with uranyl acetate and lead citrate. Sections were examined and imaged with a transmission electron microscope (Hitachi, Japan).

### 2.8 Small RNA sequencing

Total RNA was extracted from nerve tissue frozen in liquid nitrogen (n = 3). Small RNA sequencing analysis was conducted at the Novogene Biological Company (China). Thereafter, RNA purity was checked using the NanoPhotometer® spectrophotometer (IMPLEN, USA). A total amount of 3 μg total RNA per sample was used as input material for the small RNA library. Sequencing libraries were generated using NEBNext® Multiplex Small RNA Library Prep Set for Illumina® (NEB, USA.) following manufacturer’s recommendations. Next, index codes were added to assign sequences to each sample. The first strand cDNA was synthesized using M-Mu LV Reverse Transcriptase. PCR amplification was performed using LongAmp Taq 2X Master Mix, SR Primer for Illumina and index (X) primer. PCR products were purified on an 8% polyacrylamide gel (100 V, 80 min). DNA fragments corresponding to 140–160 bp (the length of small noncoding RNA plus the 3′ and 5′ adaptors) were recovered and dissolved in 8 μL elution buffer. At last, library quality was assessed on the Agilent Bioanalyzer 2100 system using DNA High Sensitivity Chips. Clean reads are obtained from raw data after quality control. Then, a certain range of length was chosen from clean reads to conduct all the downstream analyses. Differential expression analysis of miRNA was conducted using the DESeq R package (1.8.3). The P-values were adjusted according to the Benjamini & Hochberg method. A corrected P-value of 0.05 was set by default as the threshold for significantly differential expression. miRNAs were screened according to the following conditions: the corrected P value was < 0.05 and the log2 Fold Change was > 1.5.

### 2.9 RT-qPCR

The top 5 differentially expressed miRNAs at day 3 were verified by RT-qPCR in nerve tissue (n = 3). miRNAs were reverse-transcribed into their respective cDNAs using a stem-loop method (Vazyme, China), and they were quantitatively assessed using a SYBR Green I–based real-time PCR kit (Vazyme, China). (Primers list is shown in Supplementary Table1).

### 2.10 Cell transfection

Cell models with altered miR−134–5p expression were established using miR−134–5p mimics and inhibitors. MiR−134–5p mimic, miR−134–5p inhibitor, and their respective negative controls were purchased from RiboBio (Guangzhou, China) and transfected using Lipofectamine 3000. After 12 h of culture, Schwann cell (RSC, ATCC CRL−2765) were assigned to the following groups: miR−134–5p mimic, mimic negative control (mimic NC), miR−134–5p inhibitor, inhibitor negative control (inhibitor NC), and an untreated control group (received only the transfection reagent as a blank control). Then Schwann cell models with high or low miR−134–5p expression levels obtained. miR−134–5p levels were verified by RT-qPCR.

### 2.11 Cell migration assay

Schwann cell prepared as described above (miR−134–5p mimic, mimic NC, inhibitor, inhibitor NC, and control groups) were seeded into the upper chambers of Transwell inserts at a density of approximately 1 × 10^6^ cells per chamber. Complete medium fortified with 20% FBS was added to the lower chambers as the chemoattractant. After 24 h of incubation, cells were fixed for 20 min in cell fixative and stained with crystal violet. The number of migrated cells onto the lower surface of the membrane was counted under a light microscope, and 6 random fields per membrane were quantified at 200 × magnification.

### 2.12 Scratch-wound assay

RSCs were seeded into 6-well plates and cultured until a confluent monolayer formed. The medium was then replaced with serum-free medium for 12 h to synchronize the cells. Cells were subsequently divided into groups and treated with miR−134–5p mimic, mimic NC, miR−134–5p inhibitor, inhibitor NC, or PBS, and transfected using Lipofectamine 3000 according to the manufacturer’s instructions. After 24 h of transfection, a linear scratch was created in the monolayers using a sterile 200-μL pipette tip. Detached cells were gently washed away with PBS, and the medium was replaced with culture medium containing 1% FBS to minimize the influence of proliferation. Cells were kept at 37 ℃, with 5% CO₂, and at 0 h and 48 h after wounding scratch images were captured at fixed positions under a phase-contrast inverted microscope. The wound area was quantified using FIJI ImageJ.

### 2.13 CCK−8 cell proliferation assay

Cell Counting Kit−8 (CCK−8) was used to assess cell proliferation. RSCs cells in the miR−134–5p mimic, mimic NC, inhibitor, inhibitor NC, and control groups were seeded into 96-well plates at 5000 cells per well and cultured in a cell incubator. Cell proliferation was measured at 12 h, 24 h and 36 h later. Two hours before each time point, 10 μL of CCK−8 solution was added to each well. The absorbance at 450 nm (OD₄₅₀) was then measured using a microplate reader.

### 2.14 Statistical analysis

Data groups are presented as mean ± standard deviation (SD). Graphs were generated using GraphPad Prism 5.0 (GraphPad Software, Inc., USA). Statistical analyses were conducted using SPSS version 29.0 (IBM Corp., Armonk, NY, USA). Normal data distribution was checked using Kolmogorov-Smirnov test. One-way analysis of variance (ANOVA) coupled with a post-hoc Student Newman Keuls test served to compare differences among groups at each time point. A p value < 0.05 was considered statistically significant.

## 3 Results

### 3.1 SCI is associate with a post-PNI blunted responses in the distal sciatic stump

Peripheral nerves have a strong intrinsic regenerative capacity. Following injury, the peripheral nerve distal stump rapidly mounts an acute stress response, characterized by the activation of inflammatory cascades aimed at clearing the tissue debris and creating a permissive microenvironment for axonal regrowth. Macroscopically, this is often accompanied by nerve trunk edema (Grosu-Bularda et al., 2025). To assess the impact of SCI on this response, we established PNI and SCI+PNI models in Sprague–Dawley rats and examined morphological changes in the distal sciatic nerve stump over time.

From day 0 to days 3 and 7, the distal sciatic stump gradually softened and developed marked edema in the PNI group, whereas no obvious edema was observed and only a mild tissue softening was present at day 7 in the SCI+PNI group (Fig. 1b). Consistently, H&E staining showed that at day 0, nerve fibers in both groups were neatly and densely arranged. At day 3, fibers became edematous and disorganized in the PNI group, whereas they remained orderly in the SCI+PNI group, though appearing less compact than at day 0. At day 7, the nerve fibers of the PNI group had completely lost their normal architecture, while those of the SCI+PNI group still preserved their basic structure, although they appeared more loosely arranged (Fig. 1c). These findings indicate that the distal sciatic stump mounts an appropriate acute injury response after PNI alone, but this response is blunted and delayed in the SCI+PNI group.

### 3.2 SCI is associated with delayed Wallerian degeneration of the sciatic nerve in rats

The distal peripheral nerve stump must undergo Wallerian degeneration to allow for subsequent axonal regeneration. Wallerian degeneration depends on the timely breakdown and clearance of myelin; a delayed or failed myelin clearance impairs regenerative processes (Jo et al., 2023, Li et al., 2020, Liu et al., 2019, Modrak et al., 2020, Zhao et al., 2022). To assess the effect of SCI+PNI on Wallerian degeneration, we examined myelin changes in the distal sciatic nerve stump.

**Fig. 2:**
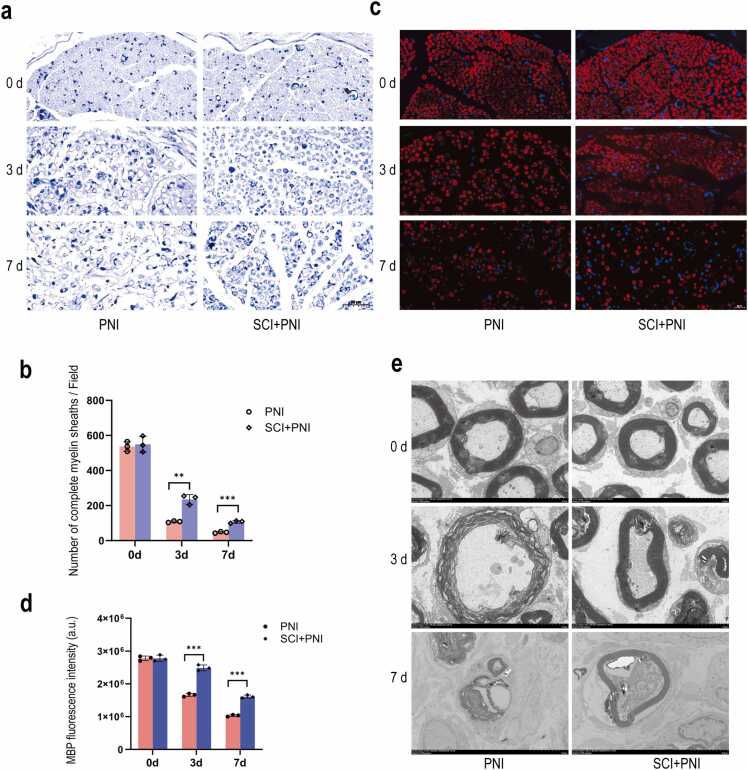
Spinal cord injury attenuates clearance of damaged myelin in the distal sciatic nerve stump (a) Toluidine blue staining of distal sciatic nerve at days 0, 3, and 7. At day 0, myelin sheaths in both groups were densely and regularly arranged with clear circular profiles. At days 3 and 7, PNI nerves showed disorganized myelin with vacuolation and progressive fragmentation, whereas SCI nerves retained more intact ring-like sheaths and fewer fragments. n = 3 per group per time point, scale bar = 20 μm. (b) Quantification of intact myelin sheaths in toluidine blue–stained sections. At day 0, counts were similar between groups. At days 3 and 7, the SCI+PNI group had significantly more intact myelin sheaths than the PNI group. n = 3 per group per time point. (c) MBP immunofluorescence staining to evaluate myelin integrity and clearance. MBP^+^ myelin is shown in red, and nuclei are counterstained with DAPI (blue). At day 0, myelin rings were intact in both groups. At day 3, most myelin in the PNI group had collapsed into debris, whereas in the SCI+PNI group the majority of sheaths remained ring-like with stronger MBP signal. At day 7, myelin was largely cleared in the PNI group, while the SCI+PNI group still showed mixed intact rings and debris with higher MBP signal. n = 3 per group per time point, scale bar = 20 μm. (d) Quantification of MBP fluorescence intensity using Fiji ImageJ. At day 0, MBP fluorescence intensity did not differ between the two groups. At days 3 and 7, MBP intensity was significantly lower in the PNI group than in the SCI+PNI group. n = 3 per group per time point.a.u., arbitrary units. (e) Transmission electron microscopy of myelin ultrastructure at the distal sciatic nerve stump. At day 0, both groups showed uniform myelin with compact dense and light layers. At day 3, SCI nerves displayed mainly deformed but ring-like sheaths with mild loosening, whereas PNI nerves showed marked lamellar separation and vacuolar degeneration. At day 7, SCI nerves exhibited mild lamellar separation, while PNI myelin had completely collapsed and disintegrated into debris. n = 3 per group per time point, scale bar = 5 μm. Data are presented as mean ± SD. **p < 0.01, ***p < 0.001.

Toluidine blue staining showed that at day 0, myelin sheaths in both groups were densely and regularly arranged, with clear circular rings and no myelin debris. At days 3 and 7, myelin in the PNI group became disorganized due to vacuolar changes, and ring-like myelin progressively fragmented. Conversely, myelin in the SCI+PNI group remained relatively better organized with more abundant intact rings and fewer fragments (Fig. 2a). Quantification of intact myelin sheaths showed that at day 3, counts were 107.67 ± 4.51 in the PNI group and 235.00 ± 27.04 in the SCI+PNI group (*P* = 0.001); at day 7, counts were 46.67 ± 4.51 and 106.33 ± 7.37, respectively (*P* < 0.001) (Fig. 2b). MBP immunofluorescence was used to further evaluate myelin clearance. At day 0, myelin rings were intact in both groups. At day 3, most myelin sheaths had collapsed into debris in the PNI group, with an MBP intensity of 1657,048.47 ± 46,744.60, whereas most sheaths retained their ring-like morphology in the SCI+PNI group, with an intensity of 2485,815.68 ± 89,174.45 (*P* < 0.001). At day 7, MBP intensity was 1032,071.18 ± 16,099.56 in the PNI group and 1608,610.70 ± 48,106.58 in the SCI+PNI group (*P* < 0.001) (Fig. 2c, d), indicating that a more efficient myelin clearance had occurred in the PNI group.

TEM analysis further confirmed these differences in myelin clearance. At day 0, myelin sheaths in both groups exhibited uniform circular profiles with clearly defined dark and clear layers. At days 3 and 7, myelin exhibited only a mild deformation and lamellar separation in the SCI+PNI group, whereas it showed a marked lamellar separation, loosening, vacuolar degeneration and, at day 7, complete collapse into debris in the PNI group (Fig. 2e). Altogether, these findings indicate that myelin fragmentation and clearance proceed efficiently after PNI but are markedly delayed after SCI+PNI, suggesting that SCI impairs the PNI-driven Wallerian degeneration in the distal sciatic nerve and hence may hinder peripheral nerve regeneration.

### 3.3 SCI is associated with reduced angiogenesis in post-PNI nerve stumps

Polarized blood vessels bridging the gaps between nerve stumps are essential for peripheral nerve regeneration, guiding axons across the gap between the proximal and distal nerve ends (Cattin et al., 2015). To investigate the effect of SCI+PNI on angiogenesis during peripheral nerve regeneration, we performed CD31 immunohistochemical staining in the injured nerve tissues. CD31 (PECAM−1) is an endothelial cell marker commonly used to evaluate vascular density and angiogenesis.

**Fig. 3:**
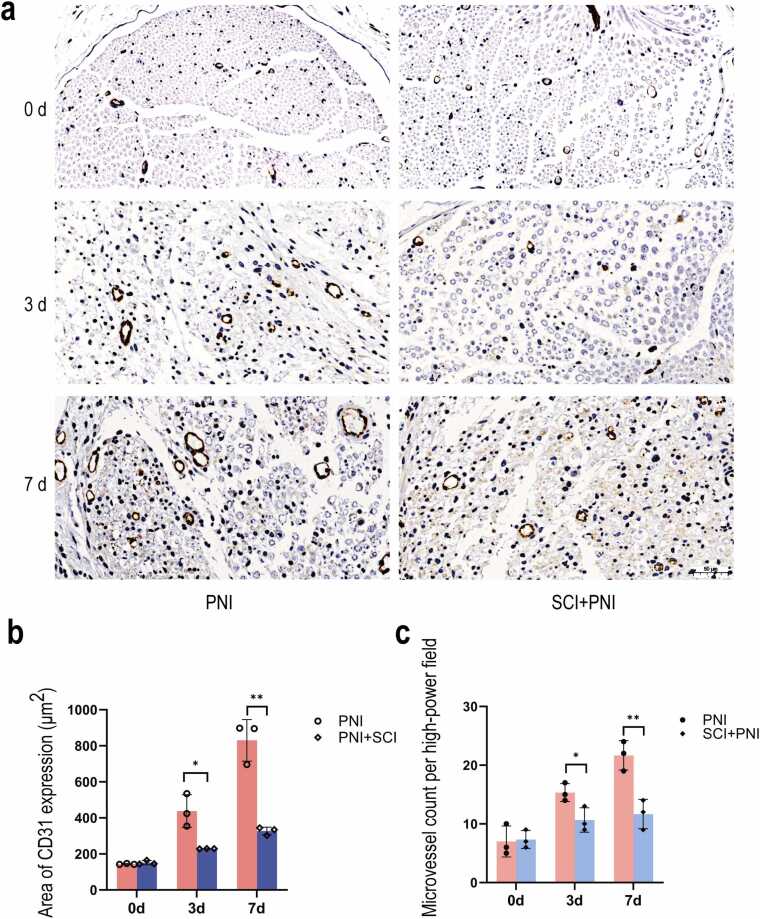
Spinal cord injury is associated with reduced angiogenic responses in the distal sciatic nerve stump(a) CD31 immunohistochemical staining of the distal sciatic nerve stump at days 0, 3 and7. At day 0, CD31^+^ endothelial cells were comparable in the PNI and SCI+PNI groups. In the PNI group, CD31^+^ ring-like structures markedly increased at day 3 and further increased in number and caliber at day 7. In the SCI+PNI group, CD31^+^ vascular rings gradually increased at day 3 and 7 but remained fewer and smaller than those in the PNI group at the corresponding time points. n = 3 per group per time point, scale bar = 50 μm. (b) Quantitative analysis of CD31^+^ area using FIJI ImageJ. At day 0, CD31⁺ area was similar was similar between the two groups (*p* = 0.343). At day 3, CD31⁺ area was significantly higher in the PNI group than in the SCI+PNI group (*p* = 0.016). At day 7, CD31 expression further increased in the PNI group, whereas only a modest increase was observed in the SCI+PNI group; CD31 expression remained significantly higher in the PNI group (*p* = 0.002). n = 3 per group per time point. (c) Quantification of CD31⁺ vascular rings per high-power field. A vascular ring was defined as a CD31⁺ structure lined by endothelial cells and containing a clearly identifiable lumen. At day 0, the number of vascular rings did not differ between the two groups (*p* = 0.859). At day 3, the PNI group showed a significantly greater number of vascular rings compared with the SCI+PNI group (*p* = 0.035). At day 7, this difference became more pronounced (*p* = 0.008). Data are presented as mean ± SD. *p < 0.05, **p < 0.01. n = 3 per group per time point.

At day 0 after surgery, the CD31^+^ endothelial cell (brown staining) was comparable between the groups (PNI group: 143.75 ± 3.04 μm²; SCI+PNI group: 151.17 ± 11.57 μm²; *P* = 0.343). At day 3, both the CD31^+^ area and the lumen diameter of ring-like vessels were greater in the PNI group than in the SCI+PNI group (PNI group: 437.83 ± 90.79 μm²; SCI+PNI group: 228.83 ± 1.01 μm²; *P* = 0.016). At day 7, the PNI group exhibited more numerous CD31^+^ endothelial cells forming larger-caliber vascular rings, with a CD31^+^ area of 830.83 ± 115.69 μm², whereas the SCI+PNI group showed fewer CD31^+^ cells with smaller luminal diameters and a CD31^+^ area of 327.42 ± 21.46 μm² (*P* = 0.002 vs. PNI) (Fig. 3a, b). In addition, the number of CD31‑positive vascular rings per high‑power field did not differ between groups at day 0 (*p* = 0.859), but was significantly greater in the PNI group than in the SCI+PNI group at day 3 (*p* = 0.035) and day 7 (*p* = 0.008) (Fig. 3c). These findings indicate that a robust early angiogenesis took place in the PNI group, which was likely to promote nerve repair, whereas the formation of polarized vessels was markedly impaired in the SCI+PNI group, which suggested that following peripheral nerve injury angiogenesis was reduced in the distal sciatic nerve stumps.

### 3.4 Small RNA sequencing reveals significant downregulation of miR−134–5p and miR−142–5p in the SCI+PNI group

**Fig. 4:**
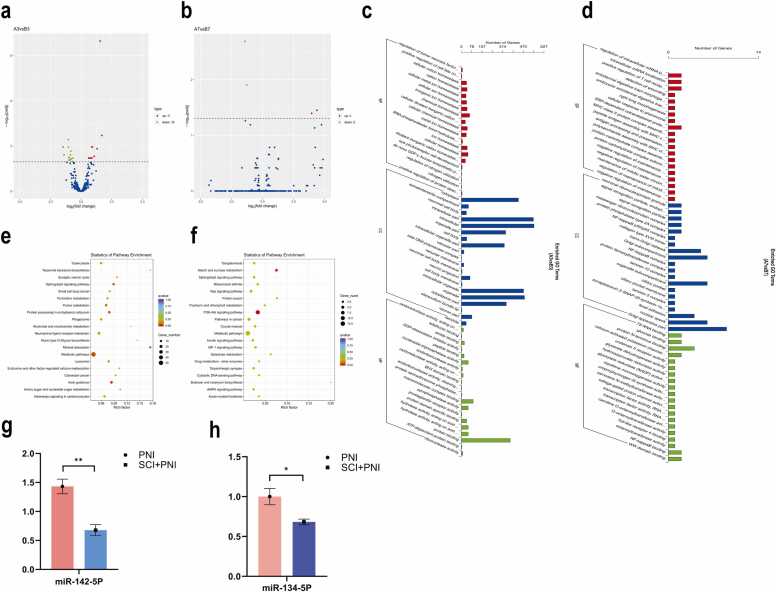
Differentially expressed miRNAs in distal sciatic nerve stumps after spinal cord injury show downregulation of miR−134–5p and miR−142–5p(a, b) Volcano plots of differentially expressed (DE) miRNAs between the PNI and SCI+PNI groups at day 3 and day 7. At day 3, 30 DE miRNAs were identified, with 13 downregulated (red) and 17 upregulated (green) in the SCI+PNI group versus the PNI group. At day 7, 6 DE miRNAs were detected, including 4 downregulated and 2 upregulated in the SCI+PNI group. (c, d) GO enrichment of predicted targets of DE miRNAs. Red, biological process (BP); blue, cellular component (CC); green, molecular function (MF). At day 3, genes were enriched in BP terms related to positive regulation of biological and cellular processes and single-tissue processes; CC terms associated with intracellular and neuron-related components and membrane-bound organelles; MF terms mainly involved protein and anion binding. At day 7, enriched BP terms were related to regulation of intracellular mRNA localization and positive regulation of T cell receptor signaling; CC terms predominantly included the Golgi apparatus, focal adhesions, and other organelles; MF terms mainly involved 7S RNA, glucose, and N-terminal protein binding. (e, f) KEGG pathway enrichment of predicted targets of DE miRNAs. At day 3, key pathways included lysosome (rno04142), axon guidance (rno04360), sphingolipid metabolism (rno00600), and DNA replication (rno03030). At day 7, enriched pathways included the PI3K–Akt signaling pathway (rno04151), metabolic pathways (rno01100), and the sphingolipid signaling pathway (rno04071). (g, h) RT–qPCR validation of miR−134–5p and miR−142–5p expression in the distal sciatic nerve stump at day 3, showing lower levels in the SCI+PNI group than in the PNI group. n = 6. A, PNI group; B, SCI+PNI group. Data are presented as mean ± SD, *p < 0.05, **p < 0.01.

To further clarify the regulatory impact of SCI+PNI on sciatic nerve regeneration, we performed small RNA sequencing of distal sciatic nerve stumps from rats in the PNI and SCI+PNI groups (RNA sequencing data have been deposited in the NCBI database, SubmissionID: SUB13715703, BioProject ID: PRJNA999489). After quality control, 30 differentially expressed miRNAs were identified in the SCI+PNI group compared with the PNI group at day 3, including 13 downregulated and 17 upregulated in the SCI+PNI group. At day 7, 6 DE miRNAs were detected, with 4 downregulated and 2 upregulated in the SCI+PNI group (Fig. 4a, b; Supplementary table 2 and 3). Target genes of these DE miRNAs were predicted using miRanda and RNAhybrid, followed by Gene Ontology (GO) and KEGG pathway enrichment analyses. At day 3, GO biological process (BP) terms with the largest number of enriched genes mainly involved positive regulation of biological processes, cellular processes in a single organism, and single-tissue processes. Cellular component (CC) terms were predominantly associated with intracellular structures, neuron-related components, and membrane-bound organelles, whereas molecular function (MF) terms were enriched in protein binding and anion binding (Fig. 4c). At day 7, the most enriched BP terms were related to regulation of intracellular mRNA localization and positive modulation of T cell receptor signaling; CC terms mainly included the Golgi apparatus, focal adhesions, and other organelles; MF terms were enriched in 7S RNA binding, glucose binding, and N-terminal protein binding (Fig. 4d). KEGG analysis showed that at day 3, the most relevant pathways were lysosome, axon guidance, sphingolipid metabolism, and DNA replication (Fig. 4e). At day 7, the main enriched pathways were the PI3K–Akt signaling pathway, general metabolic pathways, and the sphingolipid signaling pathway (Fig. 4f).

We further validated the top 5 DE miRNAs at day 3 by RT-qPCR, as this time point represents a critical window for Wallerian degeneration. The results confirmed that miR−134–5p and miR−142–5p expression patterns were consistent with the sequencing data, with lower levels in the SCI+PNI group than in the PNI group. The relative expression of miR−142–5p in the PNI and SCI+PNI groups were 1.43 ± 0.13 and 0.68 ± 0.09, respectively (*p* = 0.0045), (Fig. 4g). For miR−134–5p, its relative expression was 1.00 ± 0.10 in the PNI group and 0.68 ± 0.04 in the SCI+PNI group (*p* = 0.0262) (Fig. 4h). These findings indicate that SCI+PNI associates with a distinct miRNA expression profile in the distal sciatic nerve stump and that miR−134–5p and miR−142–5p are significantly downregulated in the SCI+PNI group, supporting their involvement in the impaired regenerative response after SCI+PNI.

### 3.5 miR−134–5p promotes rat Schwann cell migration and proliferation in vitro

Earlier studies showed that miR−142–5p and miR−134–5p are closely associate with nervous system diseases and peripheral nerve injury (Chen et al., 2016, Fu et al., 2021, Ji et al., 2019, Wang et al., 2016, Wilkerson et al., 2020, Zhang et al., 2022), and that miR−142–5p exerts neuroprotective effects and promotes nerve repair by regulating neuronal viability and apoptosis after nerve injury (Xie et al., 2020). Here, we investigated the role of miR−134–5p in peripheral nerve repair using in vitro assays of rat Schwann cell (RSC) function.

**Fig. 5:**
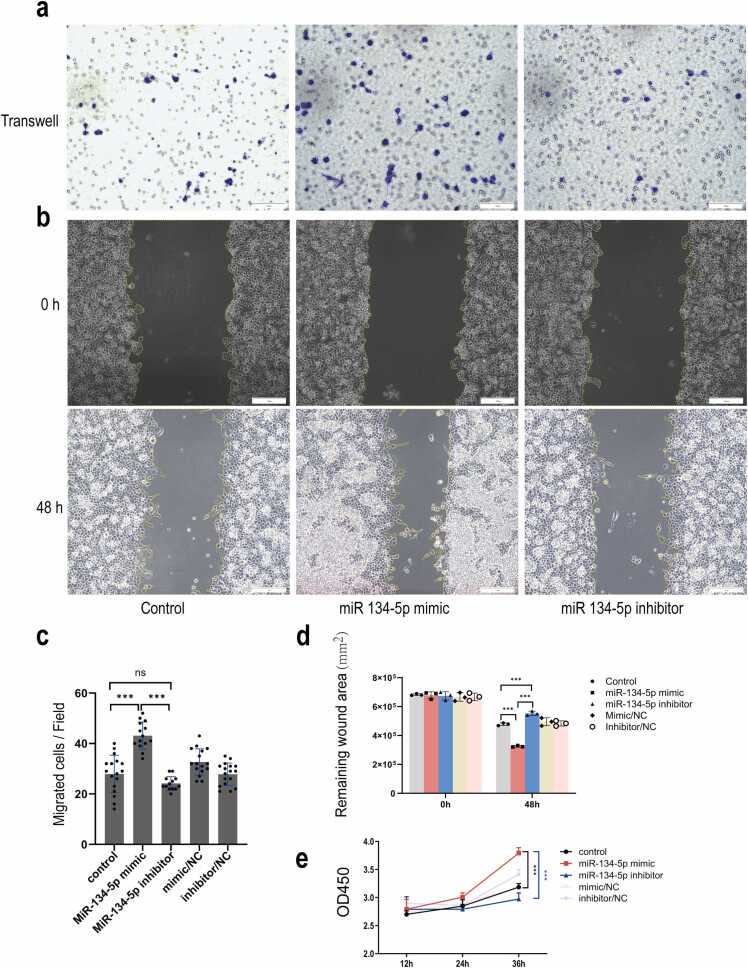
miR−134–5p promotes Schwann cell migration and proliferation *in vitro* (a, c) Transwell migration assay of rat Schwann cell (RSC). After 24 h, migrated cells on the lower surface of the membrane were stained with crystal violet. The miR−134–5p mimic group showed significantly more migrated cells than the control group (*p* < 0.001) and the miR−134–5p inhibitor group (*p* < 0.001). The difference between the control group and the miR−134–5p inhibitor group was not statistically significant (*p* = 0.495). n = 6, scale bar = 100 μm. (b, d) Scratch-wound assay to evaluate the effect of miR−134–5p on RSC migratory capacity. At 48 h after scratching, the remaining wound area (outlined by yellow dashed lines) was significantly smaller in the miR−134–5p mimic group than in the control group (*p<* 0.001) and the miR−134–5p inhibitor group (*p <* 0.001). The miR−134–5p inhibitor group showed a significantly larger remaining wound area than the control group (*p <* 0.001), indicating reduced migration. n = 6, scale bar = 100 μm. (e) CCK−8 assay of RSC proliferation. At 12 h, OD₄₅₀ values were comparable among groups. At 24 h, OD₄₅₀ in the miR−134–5p mimic group was higher than in the other groups, and by 36 h it was significantly higher than in the control and inhibitor groups. n = 6. Data are presented as mean ± SD, ***p < 0.001.

Using the Transwell migration assay, we showed that after 24 h the numbers of migrated cells were as follows: the control group, 27.94 ± 7.43; miR−134–5p mimic group, 43.14 ± 5.37; mimic negative control (mimic NC) group, 32.68 ± 5.22; miR−134–5p inhibitor group, 24.15 ± 2.73; and inhibitor negative control (inhibitor NC) group, 27.82 ± 4.45. The miR−134–5p mimic group showed a significantly higher number of migrated cells than all the other groups (*p* < 0.001). However, the miR−134–5p inhibitor group showed only a modest decrease in migrated cells compared to the control group, and this difference was not statistically significant (*p* = 0.495) (Fig. 5a, c). This may be attributable to the already low basal expression level of miR−134–5p in untreated Schwann cell, which limits the detectable effect of further inhibition in this particular assay.

In the scratch-wound assay, at 48 h the remaining wound area was 0.32 ± 0.005 mm² in the miR−134–5p mimic group; 0.48 ± 0.009 mm² in the control group (*p<* 0.001 vs. mimic); and 0.55 ± 0.015 mm² in the miR−134–5p inhibitor group (*p* < 0.001 vs. mimic). Importantly, the miR−134–5p inhibitor group also showed a significantly larger wound area than the control group (*p* < 0.001), indicating that inhibition of miR−134–5p effectively impaired directional cell migration (Fig. 5b, d). Together with the Transwell results, these data demonstrate that miR−134–5p overexpression consistently enhances Schwann cell migration, while its inhibition clearly suppresses wound closure in a two-dimensional migration setting.

Cell proliferation was assessed using the Cell Counting Kit−8 (CCK−8), with absorbance measured at 450 nm. At 12 h, OD450 values were similar among all groups. At 24 h, the OD450 value of the miR−134–5p mimic group (3.01 ± 0.08) was higher than of the control (2.85 ± 0.11) and of miR−134–5p inhibitor (2.79 ± 0.09) groups (*p* < 0.05 in both cases). At 36 h, OD450 values were respectively: mimic group, 3.79 ± 0.09; control group, 3.19 ± 0.06; and inhibitor group, 2.97 ± 0.11, (*p* < 0.001) (Fig. 5e). These results indicate that miR−134–5p overexpression significantly enhances the migratory and proliferative capacities of RSCs in vitro, suggesting that miR−134–5p promotes Schwann cell–mediated peripheral nerve repair.

## 4 Discussion

In this study, we used parallel rat models of sciatic nerve transection with and without a concurrent spinal cord transection to investigate how spinal cord injury influences the response of the distal stump of the peripheral nerve to injury. We found that SCI+PNI was associated with a blunted early reaction of the distal sciatic nerve stump, a delayed onset and slower progression of Wallerian degeneration, and a markedly reduced local angiogenesis. Small RNA sequencing revealed a distinct miRNA profile in the SCI+PNI group, with miR−134–5p and miR−142–5p significantly downregulated in the distal nerve stump, while functional assays showed that miR−134–5p promoted Schwann cell migration and proliferation in vitro. Altogether, these findings indicate that SCI+PNI disrupts key cellular and molecular events that normally support peripheral nerve regeneration. They also implicate miR−134–5p as a candidate regulator of Schwann cell–mediated nerve repair in this context.

SCI+PNI alters Wallerian degeneration in distal peripheral nerves. A major observation of this work is that the distal sciatic nerve stumps in SCI+PNI rats failed to mount the robust initial response typically seen after PNI alone. In neurologically intact animals, we observed the development of progressive edema and architectural disruption of the nerve fibers, together with an efficient fragmentation and clearance of myelin sheaths, which indicated an active stress response and ongoing Wallerian degeneration. These observations are in keeping with previous descriptions of the post-PNI early degenerative phase, in which Schwann cell rapidly de-differentiate, phagocytose myelin debris, and recruit macrophages to support local clearance and repair (Chen et al., 2019, Wei et al., 2024, Wu et al., 2025). By contrast, in the SCI+PNI group the sciatic nerve distal stumps exhibited less pronounced edema, retained the overall nerve architecture for longer, and showed a higher proportion of intact myelin rings (i.e., sheaths) at both 3 days and 7 days post-surgery, which was consistent with the quantitative toluidine blue and MBP-IF findings. MBP immunofluorescence and ultrastructural analyses confirmed that myelin breakdown and clearance were substantially attenuated in the SCI+PNI group. Although partial preservation of myelin sheaths might initially appear to be beneficial, the timely dismantling and removal of damaged myelin sheaths are preconditions for successful regeneration. Persistent myelin debris is known to inhibit axonal growth as it interferes with the formation of new myelin sheaths (Chen et al., 2019, Wu et al., 2025). Therefore, our findings support the concept that the SCI+PNI combination delays or “locks” distal nerves in a state of incomplete Wallerian degeneration, which is likely to hinder subsequent axonal regrowth and functional recovery. Mechanistically, SCI+PNI-induced immune dysfunction and autonomic imbalance may impair macrophage recruitment and activation, limit Schwann cell phenotypic conversion into repair cells, or reduce the metabolic support required for the energetically demanding degenerative processes (Martinez-Torija et al., 2025, Wang et al., 2023, Wu et al., 2025).

Impaired neoangiogenesis as a component of the altered microenvironment. Our data also highlight a substantial impairment of neoangiogenesis in the distal sciatic nerve stumps after SCI. In the PNI group, CD31 immunohistochemistry revealed a rapid and robust increase in microvessel density and caliber, consistent with the formation of new polarized vascular structures bridging the injury site and supporting the regenerating axons. Our observations are in agreement with recent work showing that a patterned wound neoangiogenesis is required for peripheral nerve repair and that the new vessels form scaffolds which guide Schwann cell cords and axonal growth (Bhat et al., 2024, Cattin et al., 2015, Wang et al., 2017, Wang et al., 2023). In contrast, CD31^+^ endothelial cells in the SCI+PNI group only showed a slight increase in numbers on days 3 and 7 post-surgery. Given that newly formed vessels deliver oxygen and nutrients and provide migrating Schwann cell and axons with physical tracks, this angiogenic disorder is likely to further hamper the regenerative potential of the peripheral nerves placed below the level of SCI (Bhat et al., 2024, Cattin et al., 2015, Wang et al., 2017). A reduced neoangiogenesis after SCI may result from altered endothelial cells responsiveness, decreased production of pro-angiogenic mediators by Schwann cell and macrophages, or systemic hemodynamic and autonomic disturbances (Martinez-Torija et al., 2025, Wu et al., 2025). Future studies measuring specific angiogenic factors and directly manipulating vascular growth will be required to clarify the relative contributions of these mechanisms.

miRNA dysregulation and Schwann cell function after spinal cord injury. By integrating miRNAs profiling with histopathology, we provide evidence that SCI+PNI-induced changes in the microenvironment of the distal nerve stump are accompanied by specific alterations in miRNA expression. At 3 days after injury, a critical window for the onset of Wallerian degeneration, we identified a panel of differentially expressed miRNAs between the SCI+PNI and PNI alone groups. Bioinformatic analyses indicated that the predicted target genes of these miRNAs are enriched in pathways related to lysosomal function, axon guidance, sphingolipid metabolism, and cell survival—all pathways closely linked to myelin clearance, Schwann cell plasticity, and neuronal regeneration (Wu et al., 2025). At 7 days post-surgery, the enriched pathways included PI3K–Akt signaling and general metabolic pathways, which further supports the notion that SCI modulates multiple aspects of the nerve regenerative program at the transcriptional and post-transcriptional levels of the nerve regenerative program. Among the differentially expressed miRNAs, miR−134–5p and miR−142–5p were significantly downregulated in the SCI+PNI group. Previous studies implicated miR−134–5p in neuropathic pain models, in which it decreases after chronic sciatic nerve injury, whereas its overexpression suppresses neuroinflammation and attenuates pain-driven behaviors (Ji et al., 2019). Similarly, miR−142–5p has been reported to regulate neuronal injury, inflammatory signaling, and blood–brain barrier integrity in models of ischemia and neuroinflammation, and that its levels correlate with the severity of neurological damage in stroke patients (Liu et al., 2025, Wang et al., 2017, Xu et al., 2024). Given the relatively well-characterized roles of miR−142–5p in neural injury, we prioritized miR−134–5p for functional validation because its function in Schwann cell biology and peripheral nerve repair remains largely unexplored. Although these studies were not performed at the peripheral nerves level, collectively they suggest that both miRNAs participate in neural injury responses and vascular immune regulation. Our in vitro experimental results show that increasing miR−134–5p levels in Schwann cell enhances their migratory and proliferative activities, while inhibition of miR−134–5p exerts the opposite effects. These behaviors are typical of repair-phenotype Schwann cell, which after injury proliferate, migrate into the distal nerve stump, clear myelin debris, and form Büngner bands (Borger et al., 2022, Chen et al., 2019, Li et al., 2023, Wei et al., 2024, Yi et al., 2016). Future studies employing Schwann cell-specific markers such as S100 or GFAP will be needed to directly correlate miR−134–5p expression levels with Schwann cell infiltration and activation in the injured nerve in vivo. Therefore, we posit that the downregulation of miR−134–5p after SCI+PNI may contribute to the subdued Schwann cell response observed in vivo, linking altered miRNA expression to impaired Wallerian degeneration and nerve regeneration. The precise targets and pathways through which miR−134–5p acts in Schwann cell remain to be clarified, but predictions based on our sequencing data and existing literature point to potential roles in cytoskeletal dynamics, growth-factor signaling and inflammatory modulation (Borger et al., 2022, Ji et al., 2019, Li et al., 2023, Martinez-Torija et al., 2025, Yi et al., 2016). The functional contribution of miR−142–5p to the impaired regenerative response after SCI+PNI warrants dedicated investigation in future studies.

While our experiments focused on the sciatic nerve, the present findings have broader implications for tissue repair in paralyzed limbs. Peripheral nerves and their associateSchwann celld Schwann cell, neuropeptides, and neurovascular interactions play critical roles in cutaneous wound healing by regulating local immune responses, stem-cell niches, and angiogenesis (Al Mamun et al., 2025, Kiya and Kubo, 2019, Xing et al., 2024). The demonstration that SCI+PNI blunts Wallerian degeneration, reduces neoangiogenesis, and alters miRNA-mediated functions in distal nerve stumps suggests that the neural component of the wound microenvironment is fundamentally undermined below the level of SCI. Although we did not directly assess cutaneous wounds, it is likely that an impaired peripheral nerve regeneration would hamper chronic wound healing in SCI patients by limiting neurotrophic support, disrupting the neural regulation of microcirculation, and altering neuroimmune crosstalk within the skin (Al Mamun et al., 2025, Kiya and Kubo, 2019, Martinez-Torija et al., 2025, Xing et al., 2024).

Several limitations of our present work should be considered. First, we focused on early time points (0, 3 and 7 days) and did not extend our observations to later stages when axonal regrowth, remyelination, and functional reinnervation occur. Thus, we cannot link the delayed Wallerian degeneration and impaired neoangiogenesis we observed here to long-term functional outcomes. Second, our work centered on global miRNA profiling and in vitro manipulation of miR−134–5p. We did not investigate the in vivo effects of modulating miR−134–5p or miR−142–5p in the injured nerve, nor did we identify specific target genes at the protein level, which limits any mechanistic insight. Third, we used a complete transection model of SCI only in young adult rats. Whether similar alterations occur in clinically more common contusion injuries, in different ages and/or sexes, or in other peripheral nerves remains to be determined. Finally, we did not examine concomitant changes in skin, muscle or microvasculature of the distal limbs and did not incorporate a cutaneous wound model, so extrapolation to human chronic wounds must be made with caution.

Despite these limitations, our study yields integrated histological, ultrastructural, vascular, and molecular evidence that spinal cord transection fundamentally alters the distal peripheral nerve response to injury. Specifically, SCI blunts the acute post-PNI reaction of distal peripheral nerves below the level of injury, delaying the onset and slowing the progression of Wallerian degeneration while markedly impairing pro-regenerative neoangiogenesis. Rather than simply reducing supraspinal input to otherwise normal peripheral nerves, SCI appears to reconfigure the capacity of these nerves to undergo timely myelin dismantling/clearance and to mount supportive vascular remodeling in the distal stump, thereby creating a less permissive microenvironment for peripheral nerve regeneration. These phenotypic changes are accompanied by distinct alterations in miRNA expression, including the downregulation of miR−134–5p (and miR−142–5p); importantly, we identify miR−134–5p as a regulator of Schwann cell proliferation and migration in vitro, two functions essential for Schwann cell–mediated nerve repair. Together, our findings highlight miRNA-mediated modulation of Schwann cell as a potential therapeutic entry point, and suggest that strategies aimed at normalizing Wallerian degeneration, enhancing neoangiogenesis, or restoring beneficial miRNA expression patterns may offer opportunities to improve peripheral nerve repair below SCI’s level and, in turn, contribute to improving wound-healing outcomes in affected patients.

## CRediT authorship contribution statement

**Daniele De Santis:** Writing – review & editing. **Anna Chiarini:** Writing – review & editing. **Ilaria Dal Prà:** Writing – review & editing. **Shene Xiao:** Investigation, Data curation. **Ubaldo Armato:** Writing – review & editing. **Fang Qi:** Investigation, Formal analysis. **Guangchao Xu:** Investigation, Formal analysis. **Lingli Jiang:** Writing – original draft, Methodology, Investigation. **Fang Zhang:** Writing – original draft, Methodology, Investigation. **Chengliang Deng:** Supervision, Funding acquisition, Conceptualization. **Zairong Wei:** Project administration, Funding acquisition, Conceptualization.

## Declaration of Generative AI and AI-assisted technologies in the writing process

During the preparation of this work the author(s) used ChatGPT in order to improve the language and readability. After using this tool/service, the author(s) reviewed and edited the content as needed and take(s) full responsibility for the content of the publication.

## Ethics Approval

All animal procedures were approved by the Institutional Animal Care and Use Committee (IACUC) of Zunyi Medical University (Approval No. KLLY (A)−2019–009) and were performed in accordance with the ARRIVE guidelines and relevant institutional and national regulations.

## Consent to Participate

Not applicable.

## Consent for Publication

All authors have approved the manuscript and agree with its submission.

## Funding

This study was supported by the Zunyi Municipal Science and Technology Cooperation Program (No. ZSKH-HZ−2024–176), Guizhou Provincial Basic Research Program (Natural Science) (No. ZK [2024]325, No. ZK [2024]297), and Guizhou Provincial Clinical Medicine Research Center - Wound Repair Research (Qiankehe Platform LCZX [2025] 005).

## Declaration of Competing Interest

The authors declare that they have no known competing financial interests or personal relationships that could have appeared to influence the work reported in this paper.

## Data Availability

The RNA sequencing data presented in this study are available in the NCBI database (BioProject ID: PRJNA999489). Other data supporting the findings of this study are available from the corresponding author upon reasonable request.
