# Supplementary material for: Spinal cord transverse section hinders Wallerian degeneration and neoangiogenesis after peripheral nerve transection: Involvement of Schwann cells' miR-134–5p
Source: IBRO Neurosci Rep. 2026 Jun 20;21:132–43. doi: 10.1016/j.ibneur.2026.06.012 (PMC13316155; doi:10.1016/j.ibneur.2026.06.012)
Supplement: Supplementary file 1 — Supplementary material [file mmc1.docx]

**Supplementary Information**

**Supplementary table 1** List of primer sequences for RT-PCR

| **Primer name** | **Sequence (5 ' to 3')** | **primer length** |
| --- | --- | --- |
| rno-miR-134-5p RT | gtcgtatccagtgcagggtccgaggtattcgcactggatacgaccccctc | 50 |
| rno-miR-134-5p F | cgcgtgtgactggttgacca | 20 |
| rno-miR-134-5p R | agtgcagggtccgaggtatt | 20 |
| rno-miR-150-3p RT | gtcgtatccagtgcagggtccgaggtattcgcactggatacgactccccc | 50 |
| rno-miR-150-3p F | cgcgctggtacaggcct | 17 |
| rno-miR-150-3p R | agtgcagggtccgaggtatt | 20 |
| rno-miR-142-5p RT | gtcgtatccagtgcagggtccgaggtattcgcactggatacgacagtagt | 50 |
| rno-miR-142-5p F | gcgcgcataaagtagaaagc | 20 |
| rno-miR-142-5p R | agtgcagggtccgaggtatt | 20 |
| rno-miR-10a-5p RT | gtcgtatccagtgcagggtccgaggtattcgcactggatacgaccacaaa | 50 |
| rno-miR-10a-5p F | cgcgtaccctgtagatccgaa | 21 |
| rno-miR-10a-5p R | agtgcagggtccgaggtatt | 20 |
| rno-miR-543-3p RT | gtcgtatccagtgcagggtccgaggtattcgcactggatacgacagaagt | 50 |
| rno-miR-543-3p F | cgaaacattcgcggtgc | 17 |
| rno-miR-543-3p R | agtgcagggtccgaggtatt | 20 |
| rno-U6 RT | gtcgtatccagtgcagggtccgaggtattcgcactggatacgacaaaata | 50 |
| rno-U6 F | agagaagattagcatggcccctg | 23 |
| rno-U6 R | atccagtgcagggtccgagg | 20 |

* Note: RT in primer name indicates reverse transcription primer, F is forward primer and R is backward primer.

**Supplementary table 2** Differential expression of miRNA top5 (up and down) between SCI group and PNI group at day 3 after nerve injury.

**Table 2**

| **sRNA** | **A_readcount** | **B_readcount** | **log_2_FC** | **pval** | **padj** | **Expression trend** |
| --- | --- | --- | --- | --- | --- | --- |
| rno-miR-10a-5p | 51167.58 | 104777.8 | -0.94274 | 0.000106 | 0.013048 | up |
| rno-miR-203a-5p | 102.2826 | 284.5554 | -1.1876 | 0.000513 | 0.023805 | up |
| rno-miR-182 | 3354.152 | 6864.588 | -0.91654 | 0.000729 | 0.023946 | up |
| rno-miR-9a-5p | 29606.71 | 52758.94 | -0.76992 | 0.000713 | 0.023946 | up |
| rno-miR-9b-3p | 29504.5 | 52524.98 | -0.76859 | 0.000729 | 0.023946 | up |
| rno-miR-142-5p | 3398.795 | 1151.92 | 1.4277 | 2.62E-09 | 1.03E-06 | down |
| rno-miR-150-3p | 136.9154 | 42.1972 | 1.3942 | 2.12E-05 | 0.004167 | down |
| rno-miR-543-3p | 627.6329 | 329.4024 | 0.85827 | 0.000132 | 0.013048 | down |
| rno-miR-134-5p | 1522.376 | 827.2934 | 0.81625 | 0.000197 | 0.01549 | down |
| rno-miR-409a-3p | 2130.485 | 1379.338 | 0.60112 | 0.000331 | 0.018637 | down |

* Note: sRNA is the miRNA name. A _ readcount and B _ readcount represent the readcount of PNI group and SCI group, respectively. *p*-adj is the corrected *p*-val. Expression trend indicates the relative expression trend of this gene (SCI vs. PNI).

**Supplementary table 3** Differential expression of miRNA between SCI group and PNI group at day 7 after nerve injury

| **sRNA** | **A_readcount** | **B_readcount** | **log2FC** | **pval** | **padj** | **Expression trend** |
| --- | --- | --- | --- | --- | --- | --- |
| rno-miR-224-5p | 140.5275973 | 374.6578128 | -1.4157 | 2.42E-06 | 0.0020438 | up |
| rno-miR-672-5p | 95.18235076 | 229.3071177 | -1.2679 | 4.42E-05 | 0.012447 | up |
| rno-miR-292-5p | 15.04755297 | 0 | 6.2851 | 1.76E-05 | 0.0074342 | down |
| rno-miR-3580-3p | 14.36106083 | 0 | 6.2181 | 0.00019899 | 0.03553 | down |
| rno-miR-741-3p | 243.8829602 | 11.74971921 | 4.3742 | 0.00021049 | 0.03553 | down |
| rno-miR-743a-3p | 32.4446701 | 2.08507868 | 3.9575 | 0.00028529 | 0.04013 | down |

* Note: sRNA is the miRNA name. A _ readcount and B _ readcount represent the readcount of PNI group and SCI group, respectively. *p*-adj is the corrected *p*-val. Expression trend indicates the relative expression trend of this gene (SCI vs. PNI).
